# Hemodynamic Study of Plaque Progression and Regression Based on Coronary CTA Imaging using Computational Fluid Dynamics Method: Preliminary Results

**DOI:** 10.1007/s12265-025-10735-7

**Published:** 2026-01-03

**Authors:** Shumin Lv, Lin Yang, Jingao Jiang, Xiaowei Liu, Wenhao Huang, Jianhua Mao, Jianjun Zhang, Tingting Chen, Lijiang Tang, Xiaochang Leng, Wei Mao, Changqing Du

**Affiliations:** 1 Zhejiang Chinese Medical University, Hangzhou, China; 2 Hangzhou Lin´an District Center for Disease Control and Prevention, Hangzhou, China; 3 Department of Geriatrics, The First Affiliated Hospital, Zhejiang University School of Medicine, Hangzhou, China; 4 Department of Cardiology, Zhejiang Hospital, Hangzhou, 310013 China; 5 Department of Cardiology, Affiliated Hospital of Hangzhou Normal University, Hangzhou, 311321 China; 6 Department of Radiology, Zhejiang Hospital, Hangzhou, China; 7 Zhejiang Key Laboratory of Integrative Chinese and Western Medicine for Diagnosis and Treatment of Circulatory Diseases, Zhejiang Hospital, Hangzhou, 310013 China; 8 School of Infrastructure Engineering, Nanchang University, Nanchang, 330031 China

**Keywords:** Coronary CTA, Hemodynamic, Plaque progression and regression, Wall shear stress, Vorticity, Helicity

## Abstract

This study evaluated coronary computed tomography angiography (CCTA)-based computational fluid dynamics (CFD) for predicting plaque dynamics in coronary artery disease. We retrospectively analyzed 22 patients (34 lesions) with paired CCTAs (mean interval 2 years). Lesions were categorized as progression (increase in diameter stenosis ≥ 5%), stable (change within − 5% to 5%), or regression (decrease in diameter stenosis ≥ 5%). Hemodynamic indices were normalized to adjacent non-diseased segments. Logistic regression identified predictors: normalized minimum wall shear stress (odds ratio (OR) = 0.38, *p* < 0.001) and maximum helicity (OR = 1.44, *p* = 0.016) predicted progression; average vorticity (OR = 0.13, *p* = 0.019) and gradient oscillatory number (OR = 0.10, p = 0.001) predicted regression. Receiver operating characteristic (ROC) analysis showed good discrimination (area under the curve (AUC) = 0.78 for progression, 0.83 for regression). These noninvasive imaging- and hemodynamic-derived markers, which were independently associated with lesion progression, may enhance coronary artery disease risk stratification by identifying high-risk plaques beyond stenosis severity, thereby informing individualized follow-up and treatment.

**Graphical Abstract:**

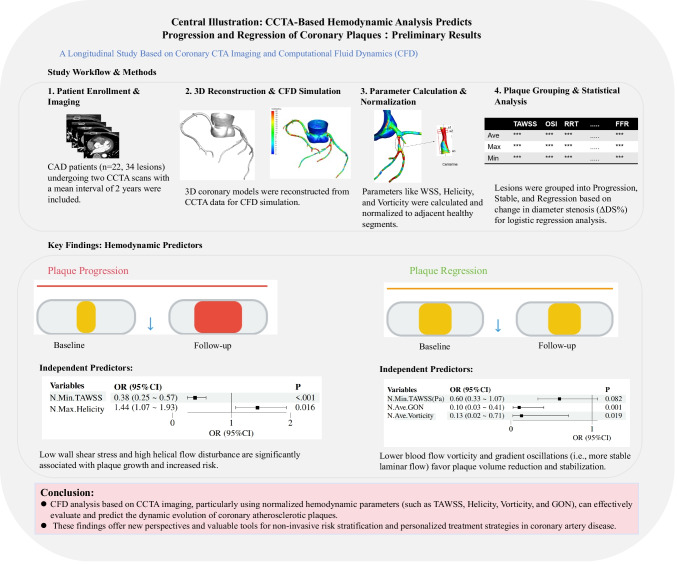

## Introduction

Coronary plaque evolution is a complex and multifactorial process involving hemodynamic alterations, inflammatory responses, and endothelial dysfunction[1, 2]. This process is closely associated with the occurrence of cardiovascular events, making the assessment of plaque progression and regression crucial for understanding the pathophysiology of coronary artery disease (CAD) and for evaluating therapeutic efficacy [3–6].

Currently, intravascular imaging techniques such as intravascular ultrasound (IVUS) and optical coherence tomography (OCT) can provide detailed morphological and compositional information on plaques. However, their invasive nature, high costs, and limited accessibility restrict their widespread clinical use [7–9]. By contrast, coronary computed tomography angiography (CCTA), as a non-invasive diagnostic tool, has been widely used for CAD diagnosis and follow-up [10–15]. Beyond visualizing coronary plaque morphology, CCTA enables quantitative analysis through computational approaches, demonstrating good consistency with IVUS in the characterization of coronary plaques [16].

In recent years, CCTA-derived fractional flow reserve (CT-FFR) has emerged as a new technology that can non-invasively assess coronary hemodynamic status, particularly for evaluating ischemic lesions [17, 18]. Studies have shown that CT-FFR is significantly associated with malignant coronary plaque characteristics and subsequent cardiac events [19]. Importantly, coronary plaques are heterogeneously distributed along the coronary tree, suggesting that local hemodynamic disturbances play a pivotal role in plaque initiation and development [20]. Moreover, plaques exhibit highly dynamic behavior, with regions showing differential progression, regression, or compositional remodeling over time [3–6].

Local hemodynamic parameters, such as wall shear stress (WSS), wall shear stress gradient (WSSG), oscillatory shear index (OSI), and relative residence time (RRT), are known to influence endothelial biology by regulating vascular tone, inflammatory signaling, and smooth muscle cell turnover [20]. Among them, abnormal WSS and its derived indices have been implicated in plaque vulnerability, rupture, acute coronary syndrome, and future adverse cardiovascular events [21–23]. Despite these findings, there remains a paucity of longitudinal studies systematically investigating how baseline WSS and its derived parameters contribute to plaque progression or regression. Furthermore, the combined effect of multiple hemodynamic factors on plaque stability remains insufficiently explored.

Therefore, the present study retrospectively analyzed patients who underwent two serial CCTA examinations to evaluate the role of baseline hemodynamic parameters in predicting subsequent plaque changes. Logistic regression analysis was performed to identify independent hemodynamic predictors of plaque stability. These findings may provide new mechanistic insights into coronary plaque dynamics and offer potential clinical value for risk stratification and individualized management of CAD.

## Methods

### Study Population

Between March 2018 and October 2021, a total of 62 patients with coronary artery disease who underwent two serial CCTA examinations were retrospectively screened. The median interval between examinations was 22 months (interquartile range, 11–32 months). Patients were excluded if they: (i) underwent coronary revascularization between the two examinations; (ii) had image artifacts or low signal-to-noise ratio that precluded reliable three-dimensional vascular reconstruction; (iii) had a time interval of less than one year between examinations; (iv) presented with other cardiovascular conditions such as aortic dissection, valvular disease, or congenital heart disease; or (v) failed to meet the technical requirements for quantitative plaque analysis and hemodynamic assessment. After applying these criteria, 22 patients were eligible for inclusion in the final analysis. Based on changes in diameter stenosis (ΔDS%), lesions were categorized into three groups: progression (ΔDS% ≥ 5%), stable (−5% < ΔDS% < 5%), and regression (ΔDS% ≤ −5%).[24, 25].

#### Coronary CTA Imaging and Post-Processing

In this study, all patients underwent CCTA using the Siemens Force CT scanner (Siemens Healthineers, Erlangen, Germany) while in a supine position. For a 64-slice CT scanner, patients were required to control their heart rate below 75 beats per minute to ensure image quality. Iodixanol (50–70 ml, flow rate 4–5 ml/s, concentration 350–370 mg/ml) was used as the contrast agent. Prospective ECG gating technology was used, and the images were processed and analyzed on the Siemens post-processing workstation.

### Plaque Measurement

Changes in stenosis degree, calcified plaque volume, non-calcified plaque volume, remodeling index, and plaque burden were calculated as the difference between follow-up and baseline measurements. Quantitative plaque analysis was performed using a dedicated workstation (syngo.via; Siemens, Germany). The following parameters were obtained: plaque length, stenosis degree, minimal lumen area, plaque total volume, calcified plaque volume, non-calcified plaque volume, remodeling index, and plaque burden. The specific calculation formulas are as follows: Stenosis degree = (proximal normal lumen area—minimum lumen area)/proximal normal lumen area × 100%; Minimal lumen area refers to the cross-sectional area of the vessel at the narrowest point of the plaque; Total plaque volume = lipid plaque volume + fibrous plaque volume + calcified plaque volume; Non-calcified plaque volume = lipid plaque volume + fibrous plaque volume. All images were independently analyzed by two experienced radiologists, with each measurement repeated three times, and the average value was used as the final result.

### Hemodynamic Calculation Process

The computation of plaque-related hemodynamic parameters from CCTA imaging was performed in four steps:

#### Anatomical Model Reconstruction and Segmentation

**Fig. 1:**
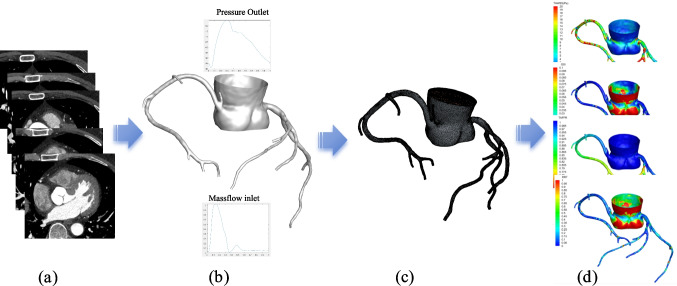
Flowchart for Calculating Hemodynamic Parameters:(**a**) CCTA image data; (**b**) Segmented 3D coronary artery model with inlet and outlet boundary conditions; (**c**) Mesh generation; (**d**) Coronary flow and pressure calculation algorithm based on the Navier–Stokes flow governing equations and distribution of hemodynamic parameters across the coronary tree. CCTA: Coronary Computed Tomography Angiography, 3D: Three-dimensional

CCTA image data were used to reconstruct three-dimensional models of the coronary arteries and left ventricle. The aorta and coronary artery tree were segmented using a combination of the fast marching algorithm and colliding fronts algorithm, allowing accurate separation from surrounding anatomical structures. Subsequently, a level-set method was applied to delineate vascular boundaries, ensuring geometric fidelity. The Marching Cubes technique, combined with automatic geometric optimization algorithms, was employed to generate the final coronary artery tree model [26]. This process is illustrated in Fig. 1a.

#### Meshing and Boundary Condition Assignment

After obtaining the coronary artery anatomical model, we perform preprocessing tasks, including hole detection, surface smoothing, and boundary refinement, before meshing. In all simulations, the inlet and outlet boundary conditions used in Fig. 1 are explicitly adopted: a prescribed mass-flow inlet based on patient-specific scaling, and reference pressure outlets [27, 28] (Fig. 1b). Patient-specific inlet boundary conditions were derived from aortic pressure and blood flow data, while myocardial mass was considered to simulate hyperemic flow (2–4 times baseline). The hyperemic state is utilized to calculate the CT-FFR values, which are measured at a location 2 to 3 cm distal to the lesion, while other hemodynamic parameters are calculated using the resting state. The vessel wall is modeled as a rigid wall with a no-slip boundary condition, and Murray's law is applied to simulate blood flow distribution in vascular branches [29–31]. Unstructured grids comprising several million elements were generated, with a maximum grid size restricted to 0.3 mm to balance numerical accuracy and computational efficiency (Fig. 1c).

#### CFD Numerical Simulation

Blood flow was modeled as an incompressible Newtonian fluid with a density of ρ = 1056 kg/m^3^ and constant viscosity [31]. The Navier–Stokes equations were solved using the finite volume method with the open-source CFD toolbox OpenFOAM. Inlet mass-flow curves (Fig. 1b), adjusted for patient-specific blood pressure, heart rate, and flow conditions, were applied at the aortic root, while outlet boundaries were defined by pressure outlet conditions [32]. The aortic outlet boundary condition satisfies the pressure outlet boundary condition. Transient calculations are performed for three cycles, with results taken from the last cycle. The calculation is considered converged when the normalized residual reaches 10^–6^.

#### Calculation of Hemodynamic Parameter Values

Hemodynamic indices were derived from the simulated flow fields. CT-FFR was calculated as the ratio of mean distal pressure to mean aortic pressure [33]. Additionally, we analyze a series of local biomechanical parameters, including wall shear stress (WSS), wall shear stress gradient (WSSG), oscillatory shear index (OSI), and relative residence time (RRT) and so on [34] (Fig. 1d). These indices reflect the endothelial shear environment and local flow dynamics, providing insights into the pathophysiological mechanisms underlying coronary atherosclerosis.

#### Hemodynamic Parameter Extraction and Normalization

**Fig. 2:**
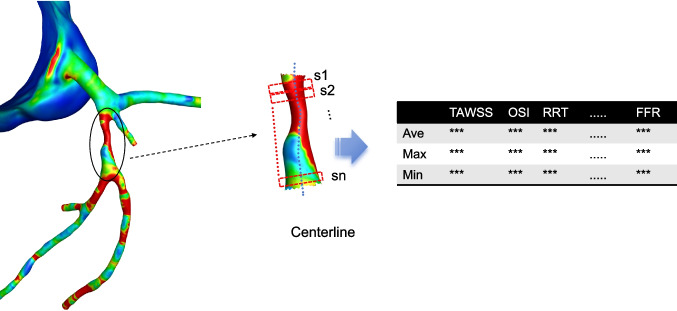
Process of acquiring hemodynamic parameters and the included parameters

For each reconstructed coronary model, hemodynamic parameters were extracted from two types of vessel segments: (i) stenotic lesion segments, defined as regions within the lesion excluding areas involving major bifurcations; and (ii) healthy reference segments, defined as proximal vessel regions without stenosis, located between the coronary artery origin and the first bifurcation. For each segment, maximum, minimum, and mean values of hemodynamic parameters were calculated (Fig. 2).

Vascular stenosis can influence blood flow not only at the lesion site but also in upstream and downstream segments within a limited range. In this study, 34 vessels yielded an average of 6–7 lesion-related segments per vessel, resulting in a total of 209 lesion segments available for analysis. Plaque progression and regression were defined according to changes in diameter stenosis relative to baseline.

Segmental analysis was performed by further subdividing each vessel into 2–4 mm sub-segments. Within each sub-segment, local hemodynamic indices, plaque morphology, and compositional attributes were quantified (Fig. 2). To reduce inter-patient variability caused by differences in boundary conditions, all hemodynamic parameters from stenotic segments were normalized to the corresponding values derived from their respective proximal healthy reference segments.

### Statistical Analysis

Clinical characteristics, plaque attributes, quantitative imaging parameters, and hemodynamic indices were collected and analyzed. As individual patients often presented with multiple plaques, each lesion was treated as an independent unit of analysis. Continuous variables with normal distribution were expressed as mean ± standard error and compared between groups using the independent-samples t-test. For non-normally distributed data, values were reported as median (interquartile range), and intergroup differences were assessed with the Mann–Whitney U test. Categorical variables were presented as frequencies or percentages and compared using the χ^2^ test.

To identify factors associated with plaque progression and regression, multivariable logistic regression models were constructed. The predictive performance of quantitative plaque parameters was further evaluated by receiver operating characteristic (ROC) curve analysis. A two-sided p-value < 0.05 was considered statistically significant. All statistical analyses were performed using R software (version 4.4.0; R Foundation for Statistical Computing, Vienna, Austria; released April 24, 2024).

## Results

### Baseline Clinical and Lesion Characteristics

Baseline clinical and lesion characteristics are summarized in Table 1. This retrospective analysis included 22 patients with coronary artery disease who underwent two serial CCTA examinations. The mean age was 62.8 ± 8.8 years, and 72.7% (16/22) were male. The body mass index (BMI) was 25.4 ± 3.0 kg/m^2^. With respect to cardiovascular risk factors, 63.6% (14/22) of patients had hypertension, 63.6% (14/22) had diabetes mellitus and 77.3% (17/22) had hypercholesterolemia. A total of 34 coronary lesions were analyzed. Of these, 50.0% (17/34) were located in the left main to left anterior descending artery (LAD), 20.6% (7/34) in the left circumflex artery (LCX), and 29.4% (10/34) in the right coronary artery (RCA). The mean minimal lumen area was 5.24 ± 2.42 mm^2^, with an average diameter stenosis of 20 ± 10%. The mean distance from the coronary ostium to the minimal lumen site was 46.2 ± 18.3 mm, and the mean lesion length was 15.3 ± 8.2 mm..

**Table 1:** Characteristics of the patients and lesions

Patients (*n* = 22)	
Age,yrs	62.77 ± 8.76
Male	16 (72.76)
Weight(kg)	70.70 ± 11.77
Height (cm)	166.55 ± 7.22
BMI(kg/m^2^)	25.35 ± 2.97
Systolic blood pressure (mmHg)	131.05 ± 16.62
Diastolic blood pressure (mmHg)	77.68 ± 8.82
Cardiovascular risk factors	
Hypertension	14 (63.64)
Diabetes mellitus	14 (63.64)
Hypercholesterolemia	17 (77.27)
Current smoker	11 (50)
Lesion characteristics (*n* = 34)	
Location	
Left main to LAD	17 (50)
LCX	7 (21)
RCA	10 (29)
Characteristics	
Minimal lumen area,mm^2^	5.24 ± 2.42
Diameter stenosis,%	20 ± 10
Distance from coronary ostium to MLA,mm	46.2 ± 18.3
Lesion length,mm	15.3 ± 8.2

Values are *n* (%) or mean ± SD

### Comparison of CCTA-Derived Quantitative Parameters

This study included 22 patients, and biochemical markers, plaque types, vessel involvement, and high-risk plaque features were systematically analyzed (Table 2).

**Table 2:** Comparison of biochemical test results,plaque types, affected blood vessels and plaque characteristics

Category	Patients (*n* = 22)
Biochemical Test Results	
Red Cell Distribution Width (%)	13.62 ± 1.72
Creatine Kinase—MB Isoenzyme (U/L)	11.73 ± 2.29
Cardiac Troponin I (ng/ml)	0.02 ± 0.01
BNP (pg/ml)	112.76 ± 328.53
Total Cholesterol(mmol/L)	4.51 ± 1.98
LDL-Cholesterol(mmol/L)	2.47 ± 0.96
HDL-Cholesterol(mmol/L)	1.09 ± 0.32
Triglycerides(mmol/L)	3.03 ± 2.90
HbA1c(%)	6.50 ± 1.45
Lipoprotein(a)(mg/L)	376.68 ± 449.35
Apolipoprotein E (mg/L)	68.83 ± 78.86
Creatinine (μmol/L)	73.32 ± 14.55
Uric Acid(μmol/L)	353.82 ± 59.81
Homocysteine (μmol/L)	18.34 ± 22.47
Mean Platelet Volume(fL)	9.53 ± 0.74
Plaque Types	
Lipid Plaque	14 (41.18)
Mixed Plaque	9 (26.47)
Calcified Plaque	11 (32.35)
Affected Blood Vessels	
Single	14 (63.64)
Two	4 (18.18)
Three	4 (18.18)
Plaque Characteristics	
Low-attenuation plaque	9 (40.91)
Napkin—ring Sign	6 (27.27)
High—risk Plaques ≥ 2	5 (22.73)

#### Biochemical Findings

For lipid-related markers, total cholesterol was 4.51 ± 1.98 mmol/L, low-density lipoprotein cholesterol was 2.47 ± 0.96 mmol/L, high-density lipoprotein cholesterol was 1.09 ± 0.32 mmol/L, triglycerides were 3.03 ± 2.90 mmol/L, lipoprotein (a) was 376.68 ± 449.35 mg/L, and apolipoprotein E was 68.83 ± 78.86 mg/L. For glucose metabolism markers, glycated hemoglobin (HbA1c) was 6.50 ± 1.45%. For kidney function and related indicators, creatinine was 73.32 ± 14.55 μmol/L, uric acid was 353.82 ± 59.81 μmol/L, and homocysteine was 18.34 ± 22.47 μmol/L.

#### Plaque Characteristics

Lipid-rich plaques were most prevalent (41.2%), followed by calcified plaques (32.4%) and mixed plaques (26.5%). Single-vessel involvement was observed in 63.6% of patients, dual-vessel in 18.2%, and triple-vessel in 18.2%. High-risk morphological features included spotty calcification in 31.8%, low-attenuation plaque in 40.9%, the “napkin-ring sign” in 27.3%, and ≥ 2 high-risk plaques in 22.7%.

### Logistic Regression Analysis of Determinants of Plaque Stability

An overview of baseline characteristics, along with a comparative analysis of normalized hemodynamic parameters, is presented in Table 3.

**Table 3:** Baseline characteristics and comparative analysis of normalized hemodynamic parameters

Variables	Total (*n* = 209)	Stable (*n* = 53)	Progression (*n* = 128)	Regression (*n* = 28)	Statistic	*P*
N.Ave.TAPressure	0.98 ± 0.04	0.98 ± 0.03	0.99 ± 0.02	0.96 ± 0.08	F = 6.89	**0.001**
N.Max.TAPressure	0.98 ± 0.04	0.98 ± 0.03	0.99 ± 0.02	0.96 ± 0.07	F = 10.67	** < 0.001**
N.Min.TAPressure	0.99 ± 0.06	1.01 ± 0.06	0.99 ± 0.04	0.96 ± 0.10	F = 7.49	** < 0.001**
N.Ave.FFR	1.00 ± 0.10	0.98 ± 0.07	1.01 ± 0.09	1.02 ± 0.16	F = 2.06	0.131
N.Max.FFR	1.02 ± 0.09	1.00 ± 0.07	1.02 ± 0.09	1.05 ± 0.14	F = 2.85	0.060
N.Min.FFR	0.99 ± 0.11	0.97 ± 0.09	1.00 ± 0.10	1.00 ± 0.19	F = 1.37	0.255
N.Ave.TAWSS	1.10 ± 0.66	1.20 ± 0.54	1.07 ± 0.73	1.03 ± 0.54	F = 0.85	0.431
N.Max.TAWSS	1.11 ± 1.05	0.99 ± 0.72	1.22 ± 1.19	0.87 ± 0.84	F = 1.82	0.165
N.Min.TAWSS	1.27 ± 1.06	1.99 ± 1.50	0.99 ± 0.69	1.20 ± 0.85	F = 19.73	** < 0.001**
N.Ave.WSSG	1.27 ± 1.33	1.14 ± 0.74	1.39 ± 1.60	0.95 ± 0.64	F = 1.60	0.204
N.Max.WSSG	1.80 ± 5.77	1.20 ± 1.41	2.27 ± 7.28	0.81 ± 0.98	F = 1.12	0.330
N.Min.WSSG	1.69 ± 1.84	1.91 ± 1.38	1.63 ± 2.11	1.55 ± 1.19	F = 0.55	0.579
N.Ave.OSI	1.81 ± 2.26	1.46 ± 2.70	2.17 ± 2.23	0.86 ± 0.45	F = 4.89	**0.008**
N.Max.OSI	2.04 ± 3.24	1.78 ± 3.37	2.39 ± 3.47	0.92 ± 0.48	F = 2.61	0.076
N.Min.OSI	6.10 ± 24.89	3.17 ± 6.27	8.02 ± 31.42	2.87 ± 2.82	F = 0.99	0.375
N.Ave.RRT	15.34 ± 117.58	1.06 ± 1.51	24.38 ± 149.76	1.03 ± 0.47	F = 0.98	0.378
N.Max.RRT	4.00 ± 13.36	1.97 ± 5.54	5.38 ± 16.56	1.55 ± 1.57	F = 1.78	0.172
N.Min.RRT	76.77 ± 743.54	1.80 ± 1.53	124.24 ± 948.47	1.69 ± 1.00	F = 0.67	0.512
N.Ave.GON	1.10 ± 0.52	1.18 ± 0.56	1.10 ± 0.51	0.92 ± 0.46	F = 2.52	0.083
N.Max.GON	0.99 ± 0.15	0.97 ± 0.15	1.01 ± 0.15	0.92 ± 0.12	F = 5.73	**0.004**
N.Min.GON	2.43 ± 3.59	3.05 ± 4.43	2.04 ± 3.15	3.06 ± 3.63	F = 2.01	0.137
N.Ave.Velocity	1.06 ± 0.43	1.20 ± 0.41	1.02 ± 0.46	1.00 ± 0.30	F = 3.40	**0.035**
N.Max.Velocity	1.04 ± 0.41	1.03 ± 0.23	1.05 ± 0.47	1.02 ± 0.39	F = 0.11	0.900
N.Min.Velocity	1.27 ± 1.07	2.01 ± 1.52	0.98 ± 0.67	1.19 ± 0.86	F = 21.07	** < 0.001**
N.Ave.Vorticity	1.10 ± 0.59	1.25 ± 0.60	1.07 ± 0.62	0.94 ± 0.34	F = 2.99	0.052
N.Max.Vorticity	1.08 ± 0.90	0.96 ± 0.65	1.17 ± 1.01	0.89 ± 0.76	F = 1.76	0.175
N.Min.Vorticity	1.41 ± 1.34	1.17 ± 0.60	1.57 ± 1.65	1.15 ± 0.20	F = 2.29	0.103
N.Ave.Helicity	0.97 ± 6.79	−0.27 ± 2.54	1.59 ± 8.37	0.46 ± 2.95	F = 1.51	0.222
N.Max.Helicity	1.75 ± 2.89	1.00 ± 0.90	2.13 ± 3.40	1.48 ± 2.56	F = 3.03	0.051
N.Min.Helicity	1.72 ± 2.79	1.14 ± 0.94	2.04 ± 3.34	1.33 ± 2.04	F = 2.31	0.102

Results for continuous variables are presented as mean ± standard deviation (SD); F: ANOVA; SD: standard deviation

#### Predictors of Plaque Progression

Logistic regression identified several hemodynamic factors associated with plaque progression (Table 4). In univariate analysis, normalized minimum time-averaged wall shear stress (Min TAWSS) (OR = 0.40, 95% CI: 0.27–0.59, p < 0.001) and normalized maximum helicity (OR = 1.38, 95% CI: 1.06–1.81, p = 0.018) were significant predictors. In multivariate analysis adjusted for confounders, normalized Min.TAWSS remained independently protective (OR = 0.38, 95% CI: 0.25–0.57, p < 0.001), with each one increase associated with a 62% reduction in progression risk. Conversely, normalized maximum helicity was a robust positive predictor (OR = 1.44, 95% CI: 1.07–1.93, p = 0.016), indicating that intensified helical flow markedly increased progression risk.

**Table 4:** Univariable and multivariable logistic regression for plaque progression (normalized hemodynamic parameters)

Variables	Univariate analysis					Multivariate analysis				
	β	S.E	Z	*P*	OR (95%CI)	β	S.E	Z	*P*	OR (95%CI)
N.Ave.TAWSS	−0.27	0.23	−1.14	0.253	0.77 (0.48 ~ 1.21)					
N.Max.TAWSS	0.24	0.18	1.30	0.193	1.27 (0.89 ~ 1.81)					
N.Min.TAWSS	−0.92	0.19	−4.69	** < 0.001**	0.40 (0.27 ~ 0.59)	−0.96	0.21	−4.70	** < 0.001**	0.38 (0.25 ~ 0.57)
N.Ave.WSSG	0.17	0.15	1.10	0.272	1.18 (0.88 ~ 1.60)					
N.Max.WSSG	0.14	0.10	1.40	0.162	1.15 (0.94 ~ 1.41)					
N.Min.WSSG	−0.07	0.08	−0.90	0.369	0.93 (0.80 ~ 1.09)					
N.Ave.OSI	0.17	0.09	1.76	0.078	1.18 (0.98 ~ 1.42)					
N.Max.OSI	0.06	0.06	1.07	0.285	1.06 (0.95 ~ 1.19)					
N.Min.OSI	0.02	0.02	0.91	0.362	1.02 (0.98 ~ 1.06)					
N.Ave.RRT	0.36	0.19	1.96	0.050	1.44 (1.00 ~ 2.07)					
N.Max.RRT	0.05	0.04	1.18	0.237	1.05 (0.97 ~ 1.15)					
N.Min.RRT	0.01	0.02	0.46	0.647	1.01 (0.97 ~ 1.05)					
N.Ave.GON	−0.31	0.31	−1.00	0.318	0.74 (0.40 ~ 1.34)					
N.Max.GON	2.10	1.16	1.82	0.069	8.17 (0.85 ~ 78.66)					
N.Min.GON	−0.07	0.04	−1.66	0.096	0.93 (0.85 ~ 1.01)					
N.Ave.Vorticity	−0.46	0.26	−1.79	0.074	0.63 (0.38 ~ 1.05)					
N.Max.Vorticity	0.28	0.20	1.38	0.168	1.33 (0.89 ~ 1.98)					
N.Min.Vorticity	0.28	0.18	1.61	0.108	1.33 (0.94 ~ 1.88)					
N.Ave.Helicity	0.07	0.04	1.54	0.123	1.07 (0.98 ~ 1.16)					
N.Max.Helicity	0.32	0.14	2.37	**0.018**	1.38 (1.06 ~ 1.81)	0.36	0.15	2.41	**0.016**	1.44 (1.07 ~ 1.93)
N.Min.Helicity	0.19	0.11	1.81	0.070	1.21 (0.98 ~ 1.49)					

Data are odds ratios (ORs) with 95% confidence intervals (CIs) and p-values. All hemodynamic parameters were normalized to the corresponding values of proximal healthy reference segments. Multivariable models were adjusted for prespecified clinical covariates (see Methods). ORs reflect change per 1-unit increase in the normalized metric (e.g., Min TAWSS per 1  Pa; vorticity per 1 s⁻^1^; helicity per m·s⁻^2^)

#### Predictors of Plaque Regression

**Fig. 3:**
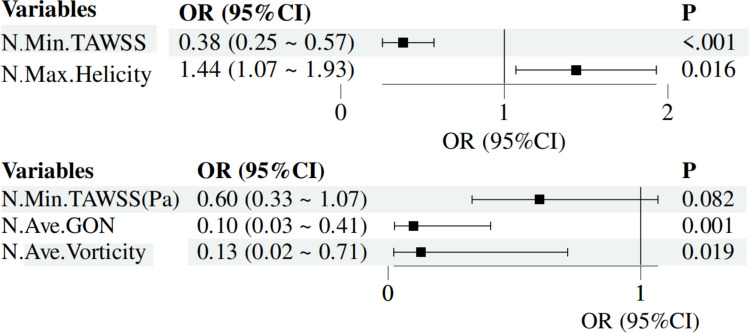
Multivariable forest plot (Plaque Progression and Plaque Regression)

In univariate analysis, none of the hemodynamic indices achieved statistical significance (Table 5). However, normalized average gradient oscillatory number (N.Ave.GON) (OR = 0.35, 95% CI: 0.13–0.93, p = 0.035) and normalized average vorticity (OR = 0.19, 95% CI: 0.05–0.67, p = 0.010) suggested a trend toward protection. In the multivariate model, both N.Ave.GON (OR = 0.10, 95% CI: 0.03–0.41, p = 0.001) and normalized average vorticity (OR = 0.13, 95% CI: 0.02–0.71, p = 0.019) emerged as significant independent predictors, indicating that higher values were associated with an 87–90% reduction in regression likelihood. Other parameters, including maximal TAWSS, WSSG, OSI, and RRT, showed no significant associations. Forest plots of multivariable models for progression and regression are presented in Fig. 3.

**Table 5:** Univariable and multivariable logistic regression for plaque regression (normalized hemodynamic parameters)

Variables	Univariate analysis					Multivariate analysis				
	β	S.E	Z	*P*	OR (95%CI)	β	S.E	Z	*P*	OR (95%CI)
N.Ave.TAWSS	−0.59	0.47	−1.27	0.205	0.55 (0.22 ~ 1.38)					
N.Max.TAWSS	−0.23	0.34	−0.68	0.498	0.79 (0.41 ~ 1.55)					
N.Min.TAWSS	−0.59	0.25	−2.32	**0.021**	0.56 (0.34 ~ 0.91)	−0.52	0.30	−1.74	0.082	0.60 (0.33 ~ 1.07)
N.Ave.WSSG	−0.40	0.37	−1.10	0.273	0.67 (0.33 ~ 1.37)					
N.Max.WSSG	−0.32	0.25	−1.26	0.209	0.73 (0.45 ~ 1.19)					
N.Min.WSSG	−0.25	0.21	−1.17	0.242	0.78 (0.52 ~ 1.18)					
N.Ave.OSI	−0.19	0.18	−1.02	0.305	0.83 (0.58 ~ 1.19)					
N.Max.OSI	−0.19	0.17	−1.12	0.262	0.82 (0.59 ~ 1.16)					
N.Min.OSI	−0.01	0.05	−0.25	0.802	0.99 (0.90 ~ 1.08)					
N.Ave.RRT	−0.02	0.19	−0.08	0.933	0.98 (0.68 ~ 1.43)					
N.Max.RRT	−0.02	0.06	−0.39	0.695	0.98 (0.87 ~ 1.10)					
N.Min.RRT	−0.06	0.18	−0.35	0.725	0.94 (0.66 ~ 1.33)					
N.Ave.GON	−1.04	0.49	−2.11	**0.035**	0.35 (0.13 ~ 0.93)	−2.29	0.71	−3.23	**0.001**	0.10 (0.03 ~ 0.41)
N.Max.GON	−2.72	1.74	−1.56	0.118	0.07 (0.00 ~ 2.00)					
N.Min.GON	0.00	0.06	0.01	0.990	1.00 (0.90 ~ 1.12)					
N.Ave.Vorticity	−1.67	0.64	−2.59	**0.010**	0.19 (0.05 ~ 0.67)	−2.07	0.88	−2.35	**0.019**	0.13 (0.02 ~ 0.71)
N.Max.Vorticity	−0.17	0.36	−0.46	0.643	0.85 (0.42 ~ 1.72)					
N.Min.Vorticity	−0.08	0.48	−0.16	0.872	0.92 (0.36 ~ 2.39)					
N.Ave.Helicity	0.11	0.09	1.13	0.257	1.11 (0.93 ~ 1.33)					
N.Max.Helicity	0.16	0.15	1.13	0.261	1.18 (0.89 ~ 1.57)					
N.Min.Helicity	0.09	0.16	0.57	0.570	1.10 (0.80 ~ 1.50)					

Data are odds ratios (ORs) with 95% confidence intervals (CIs) and p-values. All hemodynamic parameters were normalized to the corresponding values of proximal healthy reference segments. Multivariable models were adjusted for prespecified clinical covariates (see Methods). ORs reflect change per 1-unit increase in the normalized metric (e.g., Min TAWSS per 1  Pa; vorticity per 1 s⁻^1^; helicity per m·s⁻^2^)

### Predictive Performance of Normalized Hemodynamic Indices

**Fig. 4:**
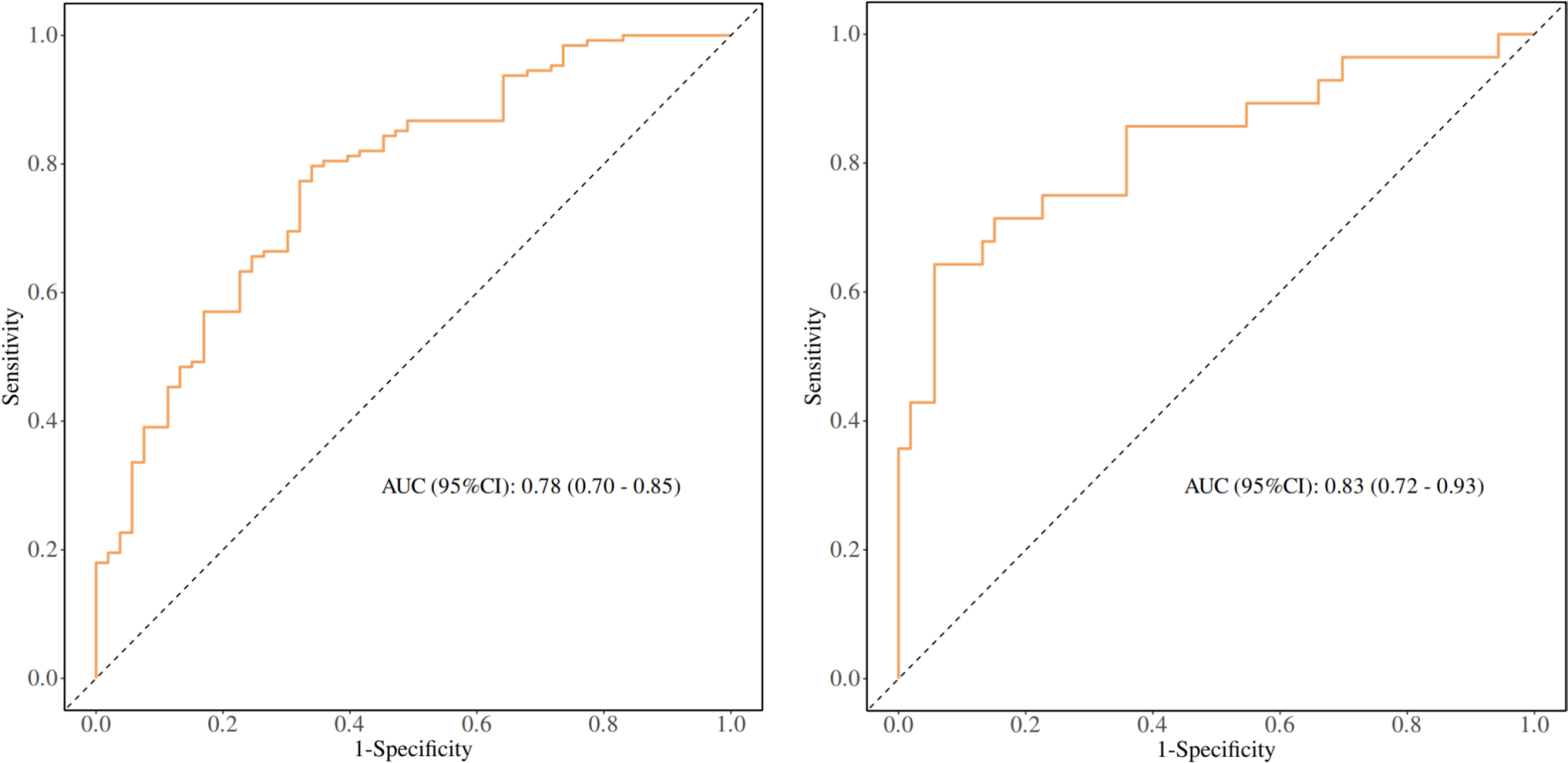
Comparison of receiver operating characteristic (ROC) curves for plaque progression (left) and regression (right) based on hemodynamic parameter prediction models

**Fig. 5:**
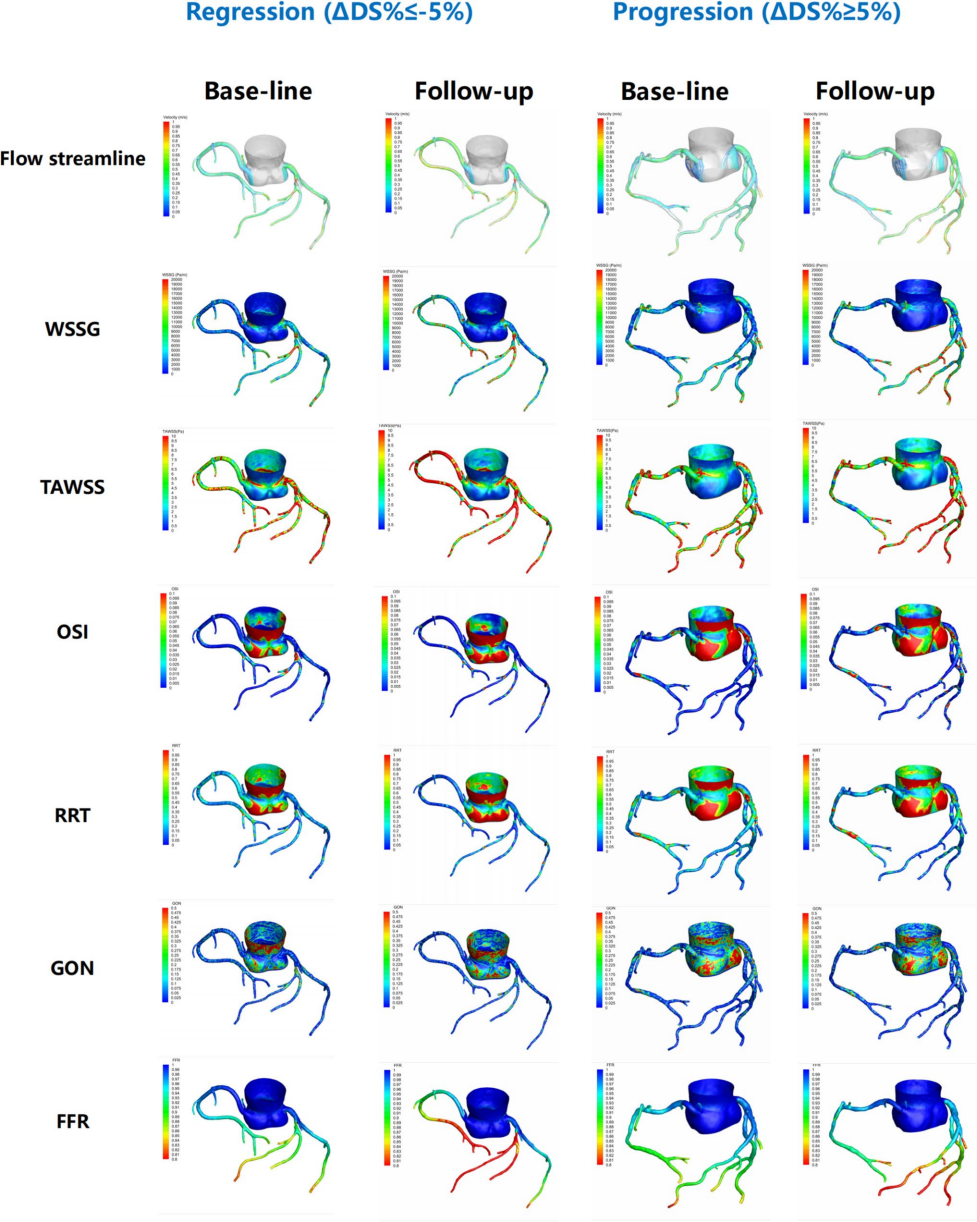
Spatial distribution maps of hemodynamic indices at baseline and follow-up for plaque progression and regression lesions

The accuracy of prediction models for plaque progression and regression is reported in Table 6, while key model evaluation metrics—ROC curves—are presented in Fig. 4, which compare the models’ predictions of plaque progression and regression (based on hemodynamic parameters). The multivariable logistic regression models showed good discrimination, with AUCs of 0.78 (95% CI: 0.70–0.85) for plaque progression and 0.83 (95% CI: 0.72–0.93) for plaque regression. For lesion-level, single-parameter ROC analyses using normalized hemodynamic indices (referenced to proximal healthy segments), the following AUCs were observed for progression: normalized minimum TAWSS, 0.73 (95% CI: 0.66–0.79) and normalized maximum helicity, 0.58 (95% CI: 0.50–0.65). For regression, normalized average gradient oscillatory number (GON) and normalized average vorticity yielded AUCs of 0.63 (95% CI: 0.51–0.73) and 0.72 (95% CI: 0.61–0.81), respectively. Representative parametric maps of hemodynamic distributions for progression and regression cases are provided in Fig. 5.

**Table 6:** Accuracy of prediction models based on hemodynamic parameters for plaque progression and regression

	AUC (95%CI)	Accuracy (95%CI)	Sensitivity (95%CI)	Specificity (95%CI)	PPV (95%CI)	NPV (95%CI)	cut off
Progression	0.78 (0.70–0.85)	0.76 (0.69–0.82)	0.66 (0.53—0.79)	0.80 (0.73—0.87)	0.57 (0.45—0.70)	0.85 (0.79—0.91)	0.693
Regression	0.83 (0.72–0.93)	0.84 (0.74–0.91)	0.94 (0.88—1.00)	0.64 (0.47—0.82)	0.83 (0.74—0.93)	0.86 (0.71—1.00)	0.497

## Discussion

This study analyzed the impact of hemodynamic parameters on plaque stability in patients with coronary artery disease. A total of 22 patients were initially enrolled, and hemodynamic parameters based on coronary computed tomography angiography (CCTA) were used to assess their coronary atherosclerotic conditions. After at least one year of regular statin therapy, patients underwent a second CCTA examination to analyze changes in plaque characteristics, thereby evaluating hemodynamic parameters influencing plaque progression and regression. Among the 34 lesions analyzed, the most common were lesions from the left main coronary artery to the left anterior descending artery (LAD). Plaque types were predominantly lipid plaques, with mixed and calcified plaques also frequently observed. In the logistic regression analysis of factors affecting plaque stability, Normalized Min.TAWSS and Normalized Max.Helicity were identified as major factors influencing plaque progression, while Normalized Ave.Vorticity and Normalized Ave.GON were key factors affecting plaque regression. ROC curve analysis demonstrated the predictive value of these parameters for plaque progression and regression. Additionally, spatial distribution maps of hemodynamic indices for progression and regression groups revealed differences between the two groups, suggesting that hemodynamic parameters may play a role in plaque progression and regression.

Non-invasive imaging techniques, such as CCTA, provide a comprehensive means of evaluating plaque morphology, composition, and hemodynamic alterations in coronary arteries. CCTA offers not only a visual representation of the coronary tree but also facilitates quantitative plaque analysis that can predict plaque progression. Hemodynamic simulations using computational fluid dynamics (CFD) models serve as invaluable tools for understanding the microenvironment of plaque progression. These models simulate blood flow in coronary artery geometries and calculate key parameters, including time-averaged wall shear stress (TAWSS) and vorticity, which are directly related to plaque stability, composition, and progression [35–37]. To understand the complex mechanisms of plaque progression and regression, it is essential to consider the hemodynamic environment in which the plaque resides. Atherosclerosis and its progression are closely associated with various hemodynamic factors, particularly wall shear stress (WSS) [38, 39]. Low WSS regions, typically found at arterial bends, bifurcations, and upstream or downstream of stenotic areas, have been shown to promote inflammatory responses, lipid accumulation, and plaque growth, thereby increasing plaque instability and rupture risk [40, 41]. In contrast, high WSS regions, while potentially inhibiting smooth muscle cell extracellular matrix synthesis and activating macrophage matrix metalloproteinases, may also lead to endothelial cell erosion and platelet activation, increasing the risk of plaque thrombosis [42].

As the frictional force exerted by blood flow on the vessel wall, WSS directly affects the physiological function of endothelial cells. Turbulent flow characterized by low WSS is commonly associated with plaque growth [41]. When Min.TAWSS is low, areas exposed to low WSS are more prone to plaque progression than those exposed to moderate or high WSS, even if the region is classified as non-plaque. Moreover, low WSS and lipid presence exhibit synergistic effects, enhancing plaque growth and leading to the highest plaque progression in lipid-rich areas exposed to low shear stress [43, 44]. Regions exposed to both low TAWSS and low multidirectional WSS demonstrate the greatest plaque progression [45]. During plaque regression, low Min.TAWSS fails to provide sufficient mechanical stimulation to activate signaling pathways such as AKT that promote cell survival and function [46], impairing macrophages' ability to clear lipids and hindering smooth muscle cell-mediated vascular wall remodeling, thereby obstructing plaque regression.

In addition to the aforementioned shared influencing parameters, Max.Helicity and Ave.GON specifically affect plaque progression and regression, respectively. Max.Helicity measures the correlation between fluid rotation and flow direction [47]. Higher Max.Helicity indicates more pronounced helical flow characteristics, and this unique flow pattern exerts distinct effects on the vessel wall. From a mechanical perspective, helical flow increases mechanical stress on the vessel wall, which, over time, damages elastic and collagen fibers, leading to structural and functional changes in the vessel wall. Simultaneously, helical flow alters the distribution and movement of substances within the plaque, facilitating local lipid accumulation and the spread of inflammatory mediators, promoting intraplaque inflammation and driving plaque progression. Models with extremely high helical flow suggest the potential existence of anti-atherosclerotic blood flow [48].

Ave.GON, as a parameter reflecting the temporal oscillations in the spatial gradient of shear stress, plays a critical role in plaque regression [49]. Higher Ave.GON values are closely associated with endothelial cell stress responses and atherosclerotic plaque instability. When Ave.GON is low, the disturbance caused by blood flow on the vascular endothelium is relatively minor [50], resulting in weaker endothelial cell stress responses. During plaque regression, low GON implies reduced mechanical disturbance on endothelial cells, facilitating sustained production of vasoprotective mediators such as nitric oxide (NO). NO exerts anti-atherogenic effects by inhibiting leukocyte adhesion, smooth muscle cell proliferation, and platelet aggregation [51]. Conversely, high GON (strong oscillations) may disrupt NO synthesis, promoting oxidative stress and inflammatory responses, thereby hindering plaque regression.

In this study, the Ave.Vorticity parameter exhibited significant effects on plaque regression. The flow disturbance reflected by Ave.Vorticity influences plaque regression. Vorticity demonstrates higher predictive accuracy for functional plaque progression than high-risk plaque (HRP) and lesion length (LL) [52]. Relatively high vorticity magnitudes are observed at the distal cap of atherosclerotic plaques, resembling turbulence in in vivo angiography. This flow generates stronger turbulence and may destabilize plaques through micro-erosion processes [53]. During plaque progression, this heterogeneity increases the probability of lipid deposition on the vessel wall, while the mechanical forces generated by disturbed flow interfere with normal endothelial cell function [54], impairing their anticoagulant and anti-adhesive properties and promoting platelet aggregation and thrombus formation [55], further facilitating plaque development.

Through hemodynamic assessment, we can analyze trends in plaque progression and regression, providing robust support for clinical decision-making. This study has certain limitations: First, there are some errors in model reconstruction and boundary condition settings, although to ensure accuracy, we invited another experienced operator to independently verify the results and minimized human error when extracting 2 mm vascular segments. Second, this study focused on baseline risk factor assessment and drug therapy, without in-depth exploration of the specific effects of medication adjustments and lifestyle changes following CCTA on plaque progression, nor detailed analysis of potential adverse cardiac events that may occur later. Third, this study employed a Newtonian, incompressible flow model with rigid walls and laminar assumptions. Transitional turbulence may arise in low-shear, recirculation/separation regions or in severe stenoses, potentially affecting hemodynamic metrics; we will consider these effects in future work. Fourth, despite the use of statins and antiplatelet therapy, LDL-C levels were at normal target; follow-up medication regimens may had no significant effect on plaque changes and are unlikely to be major confounders of the reported plaque progression/regression. We will conduct larger-scale studies with stratification by treatment intensity. Finally, this study was a small-scale observational study, and its conclusions are preliminary hypotheses that require further validation through larger-scale research.

## Conclusion

In this longitudinal CCTA-CFD study, baseline hemodynamic indices provided quantifiable, clinically meaningful prognostic signals. For progression, lower normalized Min.TAWSS predicted reduced risk (adjusted OR = 0.38; 95% CI 0.25–0.57; p < 0.001), whereas higher normalized maximum helicity predicted increased risk (OR = 1.44; 95% CI 1.07–1.93; p = 0.016). The multivariable model showed good discrimination (AUC = 0.78), with sensitivity 0.66 and specificity 0.80 at the optimal threshold (0.693). For regression, normalized average GON (OR = 0.10, 95% CI: 0.03–0.41, p = 0.001) and average vorticity (OR = 0.13, 95% CI: 0.02–0.71, p = 0.019) were independent predictors; the model achieved AUC 0.83, sensitivity 0.94, and specificity 0.64. These findings support noninvasive CFD markers for CAD risk stratification and personalized care.

## Data Availability

All data supporting the findings of this study are available within the manuscript. Additional datasets used or analyzed during the current study are available from the corresponding author on reasonable request, in compliance with institutional data-sharing policies.

## Abbreviations

CAD: Coronary Artery Disease
CCTA: Coronary Computed Tomography Angiography
CFD: Computational Fluid Dynamics
CT-FFR: Coronary Computed Tomography-based Fractional Flow Reserve
DS%: Diameter Stenosis Percentage
FFR: Fractional Flow Reserve
GON: Gradient Oscillatory Number
HRP: High-Risk Plaque
IVUS: Intravascular Ultrasound
LAD: Left Anterior Descending Artery
LCX: Left Circumflex Artery
NO: Nitric Oxide
OCT: Optical Coherence Tomography
OSI: Oscillatory Shear Index
RCA: Right Coronary Artery
ROC: Receiver Operating Characteristic
RRT: Relative Residence Time
TAWSS: Time-Averaged Wall Shear Stress
WSS: Wall Shear Stress
WSSG: Wall Shear Stress Gradient
AUC: Area Under the Curve
BMI: Body Mass Index
CI: Confidence Interval
OR: Odds Ratio
SD: Standard Deviation

## Declarations

All procedures involving human participants were conducted in accordance with the ethical standards of the institutional and national research committees and with the principles of the Declaration of Helsinki (1975) and its later amendments (2000, 2013). Informed consent was obtained from all individual participants at the time of the original imaging examinations.
